# Editorial and Miscellaneous

**Published:** 1857-12

**Authors:** 


No death from small pox has taken place in Providence (R. I.) for 17 months past. In the same city, during the last six weeks, it is estimated that there have been not less than 5000 cases of influenza—only four of which have proved fatal —*Boston Medical and Surg. Journal.*

*Iodine Injections in Uterine Hemorrhages.*—M. Dupierris, of Havanna, recommends, in the *North Amer. Med. Chir. Review*, iodine injections into the uterus in all cases "laboring under chronic uterine hemorrhage, no matter how caused, whether by an organic affection or a functional derangement." He says: "The injection is repeated, if necessary, at the end of three or four days. The next day the hemorrhage is found to have lessened; it diminishes the following day, and generally disappears on the third." "I have resorted to this method of treatment more than a hundred times, and found but one case to resist it. This patient was an old lady, who had uterine hemorrhage for many years."

*Medical Lectures the present Season.*—The Lecture Term at the Massachusetts Medical College was commenced in this city last week, with an introductory Lecture by Prof. G. C. Shattuck, which is noticed on another page. A series of clinical lectures for the coming season was commenced at the Bellevue Hospital, New York city, October 19th, with an Introductory Lecture by Dr. J. W. Francis. The lectures are to be weekly through the winter, by the following gentlemen: Profs. Alonzo Clark, B. F. Barker, and J. T. Metcalfe, and Drs. Geo. T. Elliot and James R. Wood.—Prof. St. John delivered on the same day, the Introductory Lecture at the College of Physicians and Surgeons; and Prof. Valentine Mott the one at the University Medical College.—On the evening of the 21st, Prof. J. M. Carnochan delivered the Introductory at the New York Medical College.—The 38th session of lectures at the Medical College of Ohio commenced on the 15th of October; the introductory was delivered by Prof. L. M. Lawson. —*Boston Medical and Surgical Journal.*

*A School of Medicine at Algiers.*—By a decree, dated August 4th of this year, a preparatory school of medicine and pharmacy is to be instituted in Algiers. There will be the usual number of Chairs (eight), and both the civil and military hospitals are to be opened to the pupils for clinical instruction. The school will, as far as examinations are concerned, be in connection with the Faculty of Montpellier; but the diplomas granted in Africa will entitle to practise in the French possessions of that part of the world. To commence medical studies, the young men, be they either Arab or other Mussulmen, must produce a certificate from the director of the Franco-Arabian College as to literary acquirements; the French, from the rector of the Academy of Algiers. There will be no religious restrictions.—*London Lancet.*

*Ergot as a Hæmostatic.*—At the last meeting of the Belmont (O.) Medical Society—as reported in the Western Lancet—Dr. Affleck related a case of labor preceded by severe hemorrhage, in the which the use of ergot operated favorably. He also thinks it one of the best hæmostatics we have in cases of hæmorrhage after the child is born. Dr. H. West had used the ergot to advantage in some cases of hæmorrhage, but had also used it in prior and post hæmorrhages without any apparent effect. Both gentlemen thought it had no abortive effect. Dr. Wierick thought it a good hæmostatic in cases of post delivery where the natural efforts of the uterus were present. He is now using it in a case of passive uterine hæmorrhage, as follows: Ergot, pulv., gr. v.; acetate of lead, gr. ij.; Opium, gr. i., M., three times a day. Dr. S. B. West had great confidence in its hæmostatic virtues. He had used it before and after delivery to arrest hæmorrhage, to expel the placenta, and also as a great hæmostatic.—*Boston Med. and Surg. Jour.*

*New York Academy of Medicine.*—The following, from the New York Times, is reported as part of the proceedings of the November meeting of the Academy. Dr. Van Buren read a report on Dr. Squibb's paper on chloroform, which asserted substantially that an article of chloroform is manufactured in Williamsburg not at all inferior to the best made in Europe. Dr. James, from the Committee on Materia Medica, reported in terms of glowing admiration of Borden's Concentrated Milk. Dr. Garrish presented a carcinomatous liver weighing 8 pounds, from a patient who had died aged 61 years. Dr. Barge read a paper on his improved splint. Dr. Rotton read a compiled paper, and exhibited some of the coca plant so generally chewed by the Peruvian Indians. TheFe llows took a chew all around, but exhibited none of the effects named in the paper, probably for the lack of the quicklime with which the aborigines take it.—*Boston Med. and Surgical Journal.*

